# A machine learning method to process voice samples for identification of Parkinson’s disease

**DOI:** 10.1038/s41598-023-47568-w

**Published:** 2023-11-23

**Authors:** Anu Iyer, Aaron Kemp, Yasir Rahmatallah, Lakshmi Pillai, Aliyah Glover, Fred Prior, Linda Larson-Prior, Tuhin Virmani

**Affiliations:** 1 Georgia Institute of Technology, Atlanta, 30332 USA; 2 Biomedical Informatics, University of Arkansas for Medical Sciences, Little Rock, 72205 USA; 3 Neurology, University of Arkansas for Medical Sciences, Little Rock, 72205 USA; 4 Neurobiology and Developmental Sciences, University of Arkansas for Medical Sciences, Little Rock, 72205 USA

**Keywords:** Cognitive ageing, Parkinson's disease, Machine learning

## Abstract

Machine learning approaches have been used for the automatic detection of Parkinson’s disease with voice recordings being the most used data type due to the simple and non-invasive nature of acquiring such data. Although voice recordings captured via telephone or mobile devices allow much easier and wider access for data collection, current conflicting performance results limit their clinical applicability. This study has two novel contributions. First, we show the reliability of personal telephone-collected voice recordings of the sustained vowel /a/ in natural settings by collecting samples from 50 people with specialist-diagnosed Parkinson’s disease and 50 healthy controls and applying machine learning classification with voice features related to phonation. Second, we utilize a novel application of a pre-trained convolutional neural network (Inception V3) with transfer learning to analyze the spectrograms of the sustained vowel from these samples. This approach considers speech intensity estimates across time and frequency scales rather than collapsing measurements across time. We show the superiority of our deep learning model for the task of classifying people with Parkinson’s disease as distinct from healthy controls.

## Introduction

The clinical diagnosis of Parkinson’s disease (PD) is based on 4 core clinical features: Bradykinesia, Rigidity, Rest tremor and Postural Instability. According to the UK Brain bank and Movement Disorders Society’s diagnostic criteria for PD, bradykinesia in addition to 1 of the other 3 features is needed to make a clinical diagnosis^1,2^. Strict application of clinical diagnostic criteria by experts can lead to diagnostic accuracy over 97% over the lifetime of a patient and 91.5% in the first 5 years of disease^2^. However, for non-experts, the accuracy is lower (77% over lifetime, 76% in the first 5 years)^2^. It also takes time to be this confident of the diagnosis. As a result people with PD may go many years without a diagnosis until features of their disease become more recognizable to the untrained eye. Rest tremor is one of the most easily recognized disease features but does not occur in all patients with PD. As people often attribute aging to a number of changes in gait, balance, and cognition this may also prevent people from earlier diagnosis. There are a number of supportive features that can aid in clinical diagnosis, and speech, specifically hypophonic or low amplitude speech is an early disease feature^3^. While low amplitude is the easiest speech feature to recognize in the clinic, there are other features of speech that change in people with PD (PwPD) including hoarser voice, dysarthric or slurred speech, and tachyphemic or rapid stuttering speech^4,5^. Impaired communication is present in up to 90% of people with PD, with wide variability in the degree of impairment^6^ and identification of those aspects of speech specific to this population represents a substantial body of literature^7–11^.

Speech is a complex cognitive-motor skill that is highly susceptible to degenerative changes in the vocal motor apparatus and the cognitive networks involved in speech and language production, output and comprehension^6,7^. The use of voice measurements as objective metrics to detect and track disease progression has been the focus of many studies, the majority of which have collected voice data in controlled laboratory environments. A promising avenue of research lies in development of objective metrics for detection of speech changes in PD occurring prior to onset of overt motor symptoms which could aid in earlier disease diagnosis^12^. Earlier disease diagnosis is essential to developing neuroprotective strategies, as with current diagnostic criteria at motor onset it is believed that approximately 50% of the substantia nigra pars compacta dopaminergic neurons have already degenerated^13–15^. Additionally, since recording voice samples is easy and can be accomplished in the clinic and remotely^16^, speech changes could also be used to track disease progression^17^.

While the majority of attempts to find reliable acoustic features that discriminate between PwPD and healthy controls (HC) were made using speech data recorded in a controlled environment under professional supervision, a few recent studies have explored the utility of telephonic recordings of speech^18–20^. Carron et al.^20^ analyzed the impact of uncontrolled and unsupervised recordings on the classification performance of PwPD versus HC using sustained vowel /a/ recordings from an in-house database recorded under controlled settings (30 PwPD and 30 HC). They compared their results to a subset of the same size from the mPower database^21^ recorded using a smartphone application with similar high quality (44 kHz sampling frequency) but with participants self reporting whether or not they had Parkinson’s disease. While multiple classifiers (6) were tested, the passive aggressive classifier achieved high accuracy (Area Under the receiver operating characteristic Curve or AUC between 0.8 and 0.9) using the database recorded under controlled settings but lower accuracy (AUC between 0.6 and 0.7) using the smartphone recorded database (mPower). Also, the best features differentiating the groups were different between the two databases, using any of the classifiers. In comparison, another study using parameters derived from voice captured using smartphone microphones and professional microphones showed good correlation and were deemed to be reliable in detecting pathological voices in clinical settings^22^. These conflicting results put the clinical reliability of voice recordings captured via telephonic lines or smart phone applications into question.

A substantial body of work on the value of machine learning (ML) methods to enhance classification performance using voice samples for automatic detection of PwPD has developed over the past decade^23–25^. Typically, sustained vowel phonation is used to evaluate phonatory features, while connected speech has been used to evaluate articulatory and prosodic features^23–25^. Numerous algorithms have been developed, and studies have evaluated the performance metrics between different approaches^26–28^. Recently, convolutional neural networks (CNNs) and other deep learning methods have been applied to spectrogram images of audio signals to perform speaker identification^29^ and prediction of bird species^30^ achieving good performance. However, this approach has not been applied to differentiate PwPD from HC using recordings captured via telephonic lines or smart phone applications. Hireš et al.^23^ used an ensemble of CNNs to detect PwPD in spectrogram images of vowel recordings acquired in a controlled environment, the best performance was achieved with the sustained /a/ vowel with AUC = 0.89. The authors used a multiple fine tuning (MFT) approach that consisted of three steps. First, ResNet50^31^ and Xception^32^ CNNs were trained on a large dataset of natural images (ImageNet^33^) to allow the network to learn to generate low-level image features. Weights of the CNN were fine-tuned using two datasets separately. These mediator datasets were a dataset of vowels^34^ and the Saarbruecken Voice Database (SVD)^35^ of speech recordings from 687 healthy participants and 1355 people with 71 diseases. Finally, the CNNs were trained using the PC-GITA dataset (test dataset) of 100 native Spanish-speaking subjects (50 healthy and 50 PwPD)^36^. The classification decision was made by an ensemble of these diversly fine-tuned CNNs. While the dataset of vowels is not well-characterized, both the SVD and PC-GITA datasets were recorded under controlled settings with high-quality microphones and high resolution (16-bit codeword and 44 or 50 kHz sampling frequency). It is not clear if such an approach can achieve similar performance using recordings of lower quality and lower sampling frequency such as those captured by telephones. We will show that a simpler ML approach using a state-of-the-art CNN architecture pretrained on a large library can produce equivalent classification results using lower quality voice recordings.

This study explores the reliability of voice recordings captured remotely via telephone lines in classifying PwPD and HC using machine learning approaches. We collected voice recordings of the sustained vowel /a/ from a well characterized population of 50 PwPD with a movement disorders neurologist (TV) confirmed diagnosis, and 50 HC participants. Participants called into a phone number with digital voicemail (8 kHz sampling frequency). The samples from PwPD were collected in Arkansas, a predominantly rural state, and the ability to participate from home also allowed participation from a subset of people residing in medically underserved areas that traditionally do not participate in research^16^. Such remote collection instruments in the future could allow easier tracking of disease progression and also response to novel therapies in clinical trials in PwPD. Development of a simple, cost-effective test that could be administered by a primary care physician that provides a risk assessment for potential PD could also lead to earlier referral to neurologists and even movement disorders neurologists. As voice changes may be difficult to hear by the human ear in the early stages of disease, a ML-aided classification may be more sensitive to these early changes. We therefore applied machine learning methods to classify the voice samples from our two groups. We introduce a novel application of a pre-trained Inception V3 CNN adapted to our problem with transfer-learning for the analysis of spectrogram images of the collected voice recordings. To provide a comparison in classification performance, we extracted commonly used feature vectors and applied two statistical machine learning classifiers, the random forest and logistic regression classifiers. These algorithms were chosen based on their prior use in the literature and because both approaches identify the features most significant to producing the final classification. We used cross-validation training with all ML approaches and partitioned our voice recordings randomly into training and testing sets in 100 different iterations to assess the robustness of different approaches to heterogeneity across samples.

## Results

### Study population

Voice samples from 50 PwPD and 50 HC were collected in compliance with two University of Arkansas for Medical Sciences (UAMS) Institutional Review Board (IRB) approved protocols (UAMS IRB #261021 & #273696) and in compliance with the Declaration of Helsinki. Informed consent was obtained electronically for one protocol. After pre-processing the resultant study population included 40 PwPD and 41 HC. Table 1 provides demographics of the participants whose samples were used for further analysis. These data have been made publicly available.

**Table 1:** Participant demographics.

	Healthy controls (n = 41)	Parkinson’s disease (n = 40)
Sex (male/female)	16/24	21/19
Age at enrollment (years)	47.9 ± 14.5	66.6 ± 9.0
Hoehn & Yahr stage of PD	–	2.1 ± 0.4
Disease duration (years)	–	9.5 ± 6.0

### Classification results using acoustic signal features

**Figure 1:**
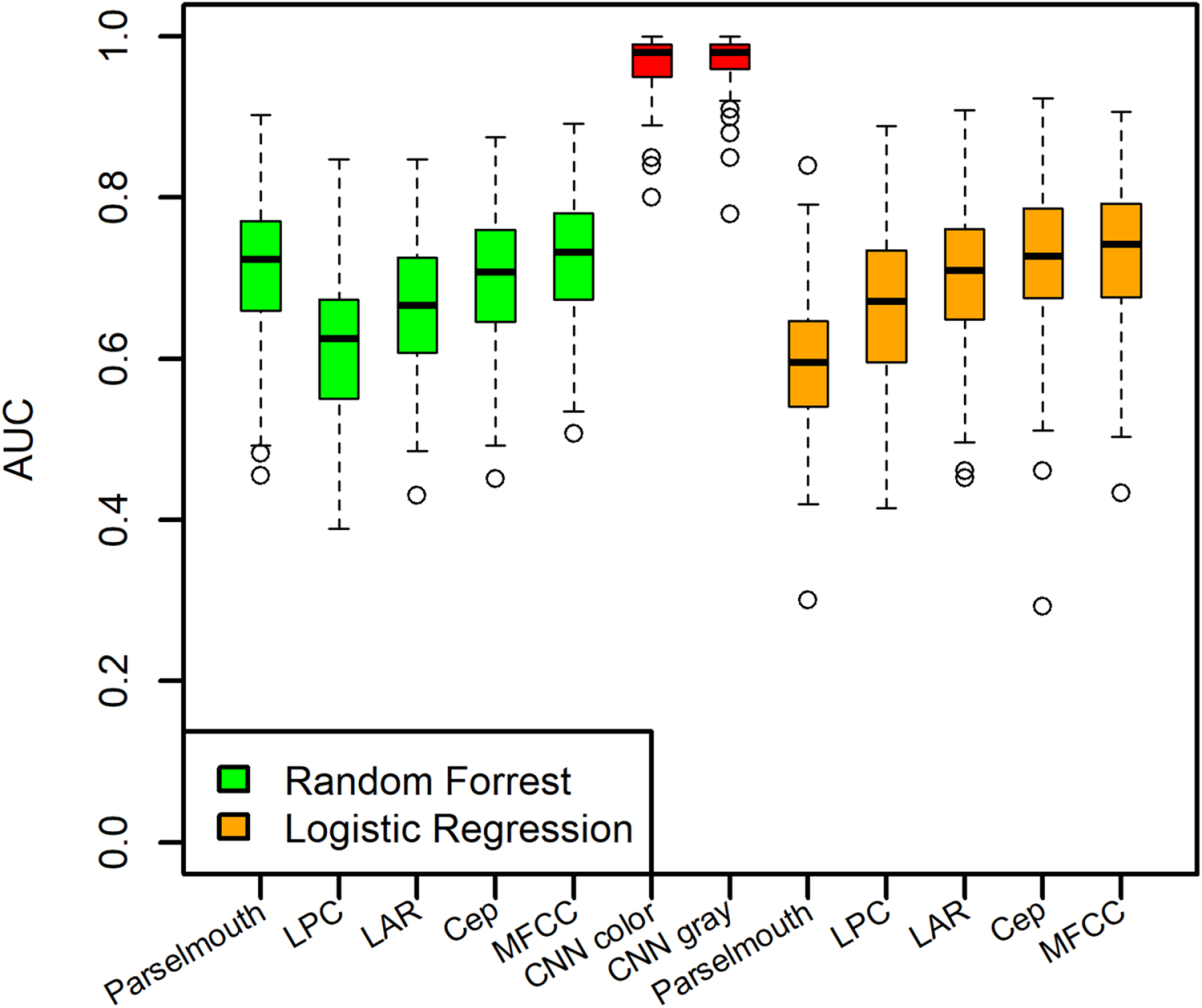
Estimated classification AUC achieved in 100 iterations using spectrograms, CNN with grayscale spectrograms, and random forest and logistic regression classifiers with acoustic signal features, and variance vectors of four spectral features (LPC, LAR, Cep, and MFCC).

Signal processing techniques were used to estimate 23 features from the sustained vowel /a/ vocalized by 40 PwPD and 41 HC. These features (identified in Fig. 1) were selected based on their common use in the literature. Logistic regression (LR) and random forest (RF) classifiers were applied with cross-validation training to estimate classification performance using the provided features (see the Methods section for details). The RF classifier outperformed the adaptive LR model (see Table 2). Figure 1 and supplementary Figure S1 respectively show boxplots of the estimated AUC values and feature importance assessed by the mean decrease Gini metric (estimated by the RF classifier) over 100 iterations. In general, standard deviation of the second formant frequency, mean of the fourth formant frequency, standard deviation of the fundamental frequency, and duration of the sustained vowel were among the most important features. The poor performance of the LR model is likely secondary to the considerable collinearity among some features; 5 types of jitter and 6 types of shimmer showed Pearson correlation coefficients > 0.95. Discarding redundancy by selecting one representative metric for jitter and one for shimmer did not improve performance.

**Table 2:** Average classification AUC using mean (m) and variance (v) vectors of the acoustic signal and spectral features or the CNN classifier using spectrogram images generated from the sustained vowel /a/.

	Acoustic Signal	LPC	LAR	Cep	MFCC	CNN
Logistic regression	0.60	0.60(m) 0.66(v)	0.56(m) 0.70(v)	0.60(m) 0.72(v)	0.50(m) 0.73(v)	0.97 (color) 0.96 (grayscale)
Random forest	0.72	0.57(m) 0.61(v)	0.56(m) 0.66(v)	0.56(m) 0.70(v)	0.57(m) 0.73(v)	

### Classification results with spectral features

Analyzing the sustained vowel /a/ uttered by 40 PwPD and 41 HC, four types of spectral feature vectors were estimated within a 32 ms sliding window with 50% overlap, resulting in a minimum of 92 windows in 1.5 s. The mean and variance vectors for each type of feature were estimated across all possible windows and used as feature vectors for classification. LR and RF classifiers were applied with cross-validation training to estimate classification performance. The mean feature vectors of all spectral features performed poorly in classifying PwPD and HC subjects (Figure S2). The variance feature vectors performed better, especially for the Cepstral Coefficients (Cep) and Mel-Frequency Cepstral Coefficients (MFCC) features (see Table 2 and Fig. 1). Supplementary Figures S3 and S4 show feature importance using the mean and variance of feature vectors from the RF analysis. Feature importance estimated by the mean decrease Gini metric is generally higher for low-order coefficients in mean feature vectors and high-order coefficients in variance feature vectors. Implementation details are available in the Methods Section.

Sex is known to have a significant effect on spectral features of speech^37,38^. We therefore examined if the sex of subjects in groups had any influence on results. A clear sex-related difference lies in the fundamental frequency (F_0_), with women’s voices 1.45–1.7× higher than males due to differences in the size of larynx^37,38^. A Chi-square test of independence between sex and group showed that the two factors are not dependent (*p*-value > 0.05) in our collected dataset.

### Classification results using a CNN

**Figure 2:**
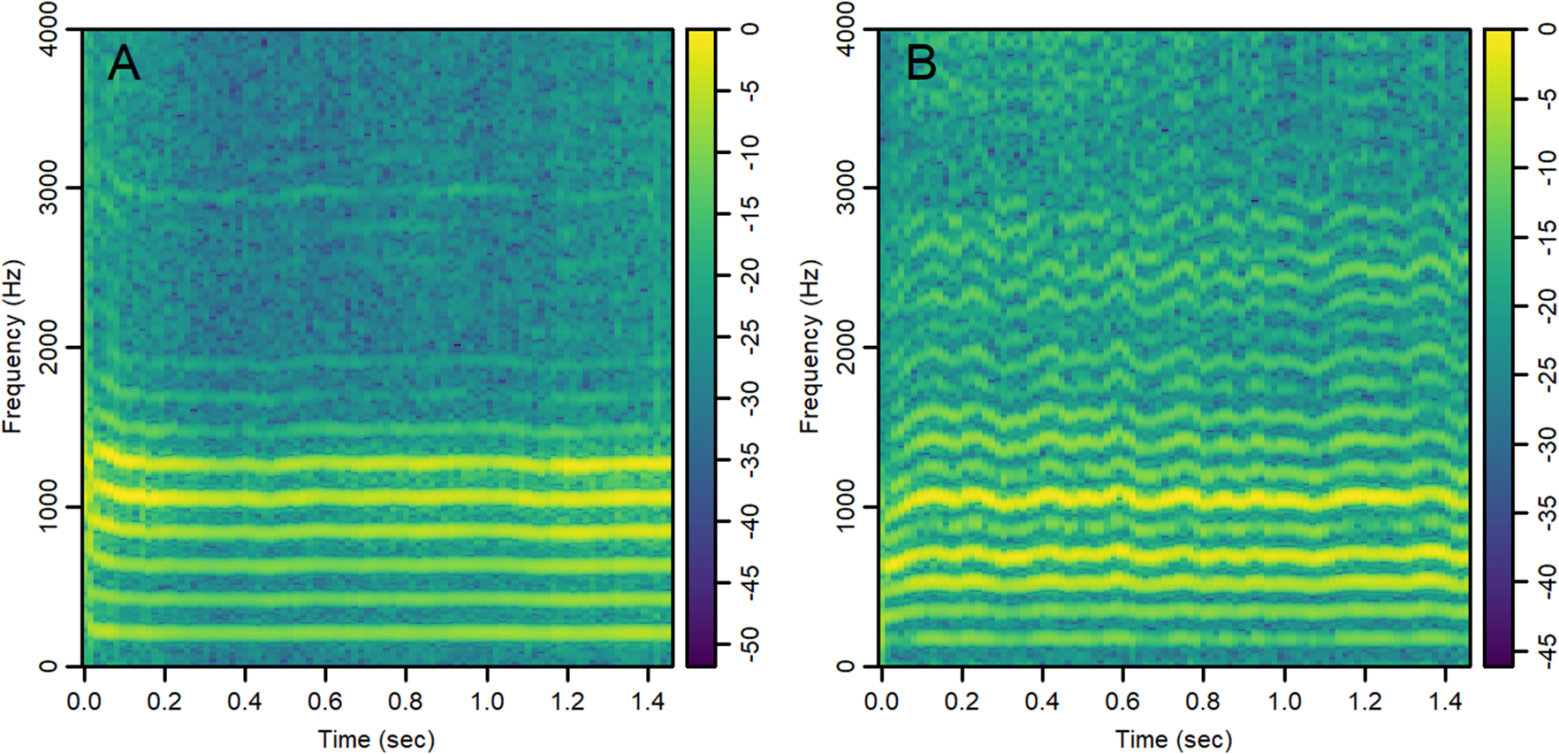
Colored spectrograms of 1.5 s of the sustained vowel /a/ uttered by selected subjects: (**A**) Healthy control (female), (**B**) Parkinson’s patient (male). The color scale represents 10 log_10_(|S|/max(|S|)), where S represents the complex numbers at the output of the FFT.

We analyzed both color (RGB) and grayscale spectrogram images of 1.5 s of the vocalized sustained vowel /a/ using an equal number of participants in the HC and PD groups (n = 40 each). Sample spectrogram images are shown in Fig. 2 for a female HC participant (panel A) and a male PD participant (panel B). Some of the horizontal bright lines represent the fundamental and formant frequencies where signal energy is concentrated around specific frequency components in the spectrum. While these examples show a larger variability in these lines across time in the PD spectrogram compared to the HC spectrogram, such a pattern was not clear in all images, and discerning the specific differences between images that contribute to the classification decision by the CNN remains a challenge. The AUC was estimated in each of the 100 iterations performed, with images randomly split into 70% training and 30% testing sets for each iteration. The achieved AUC values using both color (average AUC = 0.97) and grayscale (average AUC = 0.96) spectrogram images were found to be comparable and outperformed acoustic signal and spectral features analyzed with RF or LR classifiers (see Table 2 and Fig. 1).

## Discussion

In this study, we have collected speech recordings of the sustained vowel sound /a/ from PwPD and HC via telephonic lines and digitized recordings using 16-bit codeword representation at 8 kHz sampling frequency. We demonstrated the feasibility of using these low-resolution samples to detect speech patterns associated with Parkinson’s disease that differentiated them from healthy control subjects. We proposed the novel application of a convolutional neural network with transfer learning to analyze spectrogram images of the sustained vowel. We showed the classification performance superiority of our novel approach, which considers speech signal energy distribution across time and frequency in spectrogram images, over conventional approaches which collapse measurements across time, as utilized in the acoustic signal features or spectral features derived from linear regression models. Our results show the feasibility of using telephone-collected voice samples and the promising potential of the proposed CNN approach in detecting voice changes in PwPD.

Notably, our CNN with transfer learning approach achieved a classification performance that is comparable to the similar approach proposed by Hires et al.^23^ However, Hires et al. applied their classifier to the PC-GITA speech corpus database that is comparable in sample size to our dataset but was recorded in a noise canceling controlled environment with professional quality microphones and digitized using 44.1 kHz sampling frequency and 16-bit codewords. In contrast, our dataset was recorded using low resolution telephone recordings. This supports the approach of using easier to obtain, low-quality recordings captured by telephone lines or smartphone applications as a substitute for high-quality recordings.

We chose to explore the impact of analyzing color spectral images versus grayscale images, as the CNN architecture we employed (Inception V3) was pretrained using the ImageNet dataset which is comprised of color images. We used pretraining since our dataset is too small to train a deep learning model de-novo so we relied on transfer learning. Since color images employ 3 data channels and grayscale images only one, we wanted to ensure this difference would not negatively impact our transfer learning results. Both color and grayscale spectrogram images achieved similar AUC values in 100 iterations (Fig. 1 and Table 2).

Aging is associated with changes in both speech production and comprehension as well as hearing sensitivity leading to poorer speech recognition and comprehension^37,39–42^. Physiologically, age-related vocal changes are due to decreases in pulmonary function often associated with weakened respiratory musculature, weakening of laryngeal musculature and neuromuscular changes in facial muscles associated with articulation^41^. The clearest age-related change is in voice pitch, with F_0_ decreasing in both males and females until around 50 years of age, after which it increases in males^37^. A recent study in 500 native French speakers ranging in age from 20 to 93 addressed age and sex related changes in speech^39^. Overall, chronological age alone was found to only moderately explain the variance in analyzed speech features, although age was the best predictor of F_0_ standard deviation in both sexes. The study also replicated earlier work^37,40^ reporting increases in jitter, shimmer and F_0_ standard deviation beginning in middle age, and the increase in mean pitch in males older than 75 years^39^. In our study population, PwPD were on average 18.7 years older than HC, with the age range of the entire study population between 48 and 67. This age range does include participants in whom age-related changes in vocal features are beginning. However, including age as a covariate in a generalized linear regression model still detected a significant difference (*p*-value = 0.02) in mean F_0_ between PwPD and HC. The independent variables in the model (acoustic signal features) also did not show any significant correlation with age. This suggests that in our cohort age differences between PwPD and HC did not influence our results.

In our dataset, some of the measured features and patterns observed in spectrogram images were gender-related. However, the classification results indicate that the inter-group differences between PwPD and HC were larger than the intra-group variability due to sex, age, and individual voice characteristics within each group. Without this, such a high classification rate would not have been achievable.

### Limitations

One of the limitations of our CNN with transfer learning approach is the difficulty in determining the features or specific regions in spectrogram images that contribute to the high classification accuracy. In contrast, acoustic signal and spectral features have clear and meaningful interpretations associated with the speech excitation source (vocal folds) or tunning in the vocal cavity. Another limitation was that HC participants in the study self-reported whether they had a previous speech, neurologic, or psychiatric disorder and were not examined by a neurologist as were the PwPD. Applicability is also posed as a current limitation as our model is saved directly on a computer and is not publicly accessible.

## Conclusion

Our results show the reliability of telephone-collected voice samples, and the superiority of the proposed CNN with transfer-learning approach against the representative conventional approaches for the task detecting pathologic speech associated with Parkinson’s disease using phone-captured voice recordings of the sustained vowel /a/ under uncontrolled settings. The proposed CNN approach also shows smaller variability in classification performance when different subsets of voice recordings are used in training and testing phases. This successful novel application shows the potential of the proposed approach and the feasibility of using low-quality recordings for clinical applications. Additional development and validation of this approach may potentially enable remote monitoring of PwPD, including in rural, medically underserved communities.

## Methods

### Subjects and data collection

Voice samples for 50 PwPD and 50 HC were collected using previously published^16^ methods of collection and analysis. Briefly, participants were asked to sustain vocalization of the vowel /a/ for approximately 3 s aloud while leaving a digital voicemail. Voice was digitized using 16-bit codeword representation at 8 kHz sampling frequency.

### Data pre-processing

The resultant wav files were imported into Audacity® to remove background noise. Recorded waveforms were filtered using floor and ceiling values of 75 decibels (dB) and 300 dB respectively for males, and 100 dB and 600 dB for females. All speech signals were rescaled to the range [− 1,1]. Intervals of silence at the beginning and end of the sustained vowel sound were trimmed using a threshold level on short-time energy within a sliding window (100 samples or 12.5 ms in width). Any recording shorter than 1.5 s after trimming silent parts was omitted from the analysis. This yielded 41 HC and 40 PwPD recordings.

### Acoustic signal features

Parselmouth^43^ a Python interface to Praat^44^ was used to extract traditionally studied signal features associated with phonation in sustained vowels such as f mean and standard deviation of fundamental frequency (F0), formant frequencies, harmonics to noise ratio (HNR), jitter, and shimmer. The fundamental frequency is defined as the approximate frequency of the periodic voiced speech signal, and it measures the oscillation rate of the vocal folds. HNR is defined as the ratio of periodic and non-periodic components of the speech segment, jitter describes the fundamental frequency variation over time, and shimmer describes the variation in signal amplitudes over time. Formant frequencies represent spectral maxima that results from the acoustic resonance of the vocal tract. Mean and standard deviations of the first four formants (f1, f2, f3, and f4) were estimated and included in feature vectors. The features were estimated over the duration of the sustained ‘Ah’ sound. In total, 23 standard features were included in the analysis.

### Spectral features

Speech was analyzed in short segments within a sliding window of 32 ms (256 samples) with 50% overlap between steps. Speech signal in each segment was fitted to a linear time-invariant autoregressive model of order *p* = 10 using R package *gsignal*^45^ where 10 coefficients are sufficient to estimate the spectral envelope of a speech signal sampled at 8 kHz. The generated Yule-Walker system of linear equations was solved using the Levinson-Durbin algorithm^46^ to estimate the Linear Prediction Coding (LPC) coefficients (${a}_{1}, ... ,{a}_{10}$). A byproduct of the Levinson-Durbin algorithm is the vector of partial correlation coefficients (${c}_{1}, ... ,{c}_{10}$) which was converted to the Log-Area Ratio (LAR) feature vector using the transformation:

$${g}_{k}=log\left(\frac{1-{c}_{k}}{1+{c}_{k}}\right) , 1\le k\le p$$

Recursive calculations were performed to calculate the Cepstral Coefficients (Cep) and Mel-Frequency Cepstral Coefficients (MFCC) using R package *tuneR*^47^. Cepstral analysis deconvolves the glottal excitation signal and the vocal tract model’s impulse response in speech signal^48^. Mel-scale considers the frequency resolution of the human ear (perception) which is approximately linear below 1 kHz and logarithmic above 1 kHz. Cepstral and MFCC have generally shown good performance in speech analysis applications, including the ability to detect slight misplacements in articulators in PD^49^. The mean and variance vectors for each of the four types of coefficients (LPC, LAR, Cep, and MFCC) were calculated and used as feature vectors with logistic regression and random forest classifiers to assess the classification performance.

### Statistical machine learning classifiers

Logistic regression and random forest classifiers were used to assess the classification performance of acoustic signal and spectral feature vectors. The coefficients included in the logistic regression model were selected based on the Akaike information criterion (AIC) from the R package *MASS*^50^ and the model was trained using the R package *caret*^51^. The random forest model was built using Breiman’s algorithm^52^ as implemented in the R package *randomForest*^53^ (number of trees = 1000, number of variables randomly sampled as candidates at each split = 6, minimum size of terminal nodes = 5). The dataset was split into 70% training and 30% testing parts. The training part was subjected to threefold cross-validation to estimate the final model, and independent testing was performed using the 30% testing part. To obtain a more robust estimate of performance, the random split into training and testing parts was repeated 100 times, and the Area Under the ROC Curve (AUC) was estimated in each iteration. Feature importance was assessed using the mean decrease gini metric estimated by the RF classifier.

#### Spectrograms

We created an equal number of spectrogram images of the sustained vowel /a/ in both groups (40 PwPD and 40 HC samples) for the classification with CNN task. All recordings were at least 1.5 s in length and to make the images directly comparable, all recordings were trimmed such that only 1.5 s is considered. Spectrogram data were generated using function specgram from R package *signal* with 32 ms sliding window, 50% overlap rate, and 1024 fast Fourier transform (FFT) size. Spectrogram images show the distribution of signal energy across time and frequency axes using color intensities. The color scale represents normalized energy using 10 log_10_(|S|/max(|S|)), where S represents the complex numbers at the output of the FFT (Fig. 2). Images were created using function imagep from R package *oce*^54^ and saved in jpg file format with 600 pixels in both width and height, and 24-bit color depth. Both colored and grayscale spectrogram images were created to see if the color choice has any effect on classification performance.

#### Convolutional neural network (CNN)

A convolutional neural network is a deep learning algorithm that learns image features of relevance to the problem it is designed to solve by the application of a chain of digital filters, the parameters of which are learned. We chose the 48 layer Inception v3 CNN architecture^55^ pretrained on the ImageNet database because it has been shown to adapt successfully to medical imaging problems through transfer learning with high accuracy^56,57^. The pretrained model already extracts features that enable it to solve image classification problems. Our data set is used to refocus that ability to the specific problem of classifying spectrograms into HC and PwPD classes.

We analyzed spectrogram images of the sustained vowel /a/ in 40 PwPD and 41 HC samples for the classification task. Images were normalized to the range [0,1] and reformatted to 600 × 600 pixels. The samples were randomly split into 70% training and 30% testing parts. The random split into training and testing parts was repeated 100 times, and the AUC was estimated in each iteration (Fig. 1). Image augmentation was not applied. The original classification stage of the Inception model was replaced with four custom layers: batch normalization, dense, dropout, and a final dense layer to create a multi-layer perceptron classifier stage. Batch normalization standardizes data in between layers instead of in the raw data, which allows for run time to decrease. Dense layers execute matrix–vector multiplication when receiving input from all of the neurons in previous layers. The dropout layer prevents the models from overfitting. The model was compiled with the Adam optimizer, a learning rate of 0.001, epoch count of 10, and batch size of 4. Each run saved a model checkpoint as a ‘.h5’ file and printed the respective AUC. All 100 AUC were imported into an Excel sheet and the mean was calculated—the model achieved a 0.97 AUC for colored spectrograms and a 0.96 AUC for grayscale spectrograms.

## Supplementary Information

Supplementary Figures.

## Data Availability

Participant demographics and voice recordings are available from figshare as “Voice Samples for Patients with Parkinson’s Disease and Healthy controls”, https://doi.org/10.6084/m9.figshare.23849127. Institutional IRB and regulatory affairs decisions equate the spectrogram images created from these files to a voice print which is protected health information and cannot be publicly shared. Figure 2 is a non-computable illustration of these data and publication is permitted by the same institutional authorities.
