# Author Correction: A machine learning method to process voice samples for identification of Parkinson’s disease

**DOI:** 10.1038/s41598-026-61935-3

**Published:** 2026-08-06

**Authors:** Anu Iyer, Aaron Kemp, Yasir Rahmatallah, Lakshmi Pillai, Aliyah Glover, Fred Prior, Linda Larson‑Prior, Tuhin Virmani

**Affiliations:** 1 Georgia Institute of Technology, Atlanta, 30332 USA; 2 Biomedical Informatics, University of Arkansas for Medical Sciences, Little Rock, 72205 USA; 3 Neurology, University of Arkansas for Medical Sciences, Little Rock, 72205 USA; 4 Neurobiology and Developmental Sciences, University of Arkansas for Medical Sciences, Little Rock, 72205 USA

Correction to: *Scientific reports* 10.1038/s41598-023-47568-w, published online 23 November 2023

This Article contains errors in Figure 1, Table 2, the Results section and the subsection ‘Statistical machine learning classifiers’ due to an implementation issue within the convolutional neural network (CNN) analysis pipeline used to generate the classification results.

The correct Figure 1 and accompanying legend appear below.

**Figure 1:**
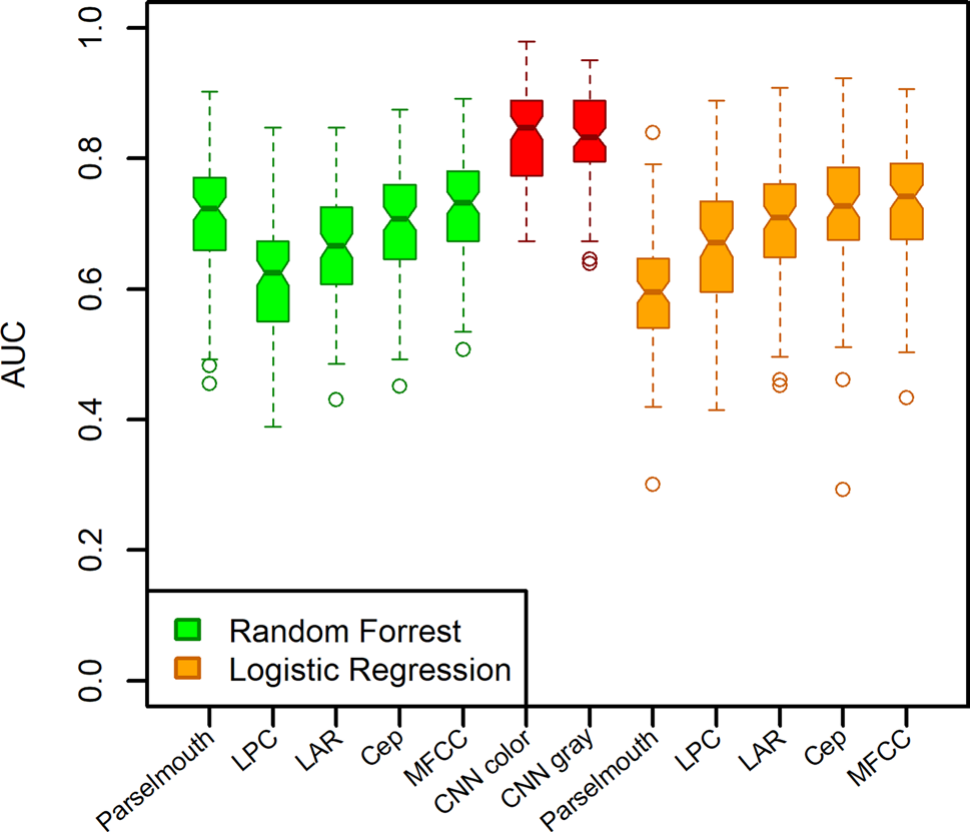
Estimated classification AUC achieved in 100 iterations using spectrograms, CNN with grayscale spectrograms, and random forest and logistic regression classifiers with acoustic signal features, and variance vectors of four spectral features (LPC, LAR, Cep, and MFCC).

The correct Table 2 and accompanying legend appear below.

**Table 2:** Average classification AUC using mean (m) and variance (v) vectors of the acoustic signal and spectral features or the CNN classifier using spectrogram images generated from the sustained vowel /a/.

	Acoustic Signal	LPC	LAR	Cep	MFCC	CNN
Logistic Regression	0.60	0.60(m) 0.66(v)	0.56(m) 0.70(v)	0.60(m) 0.72(v)	0.50(m) 0.73(v)	0.83 (color) 0.83 (grayscale)
Random Forest	0.72	0.57(m) 0.61(v)	0.56(m) 0.66(v)	0.56(m) 0.70(v)	0.57(m) 0.73(v)	

In the Results section under the subheading ‘Classification results using a CNN’,

“The achieved AUC values using both color (average AUC = 0.97) and grayscale (average AUC = 0.96) spectrogram images were found to be comparable and outperformed acoustic signal and spectral features analyzed with RF or LR classifiers (see Table 2 and Fig. 1).”

should read:

“The achieved AUC values using color or grayscale spectrogram images were found to be comparable (average AUC = 0.83) and outperformed acoustic signal and spectral features analyzed with RF or LR classifiers (see Table 2 and Fig. 1).”

In the subsection ‘Statistical machine learning classifiers’ under the subheading ‘Convolutional neural network (CNN)’,

“All 100 AUC were imported into an Excel sheet and the mean was calculated—the model achieved a 0.97 AUC for colored spectrograms and a 0.96 AUC for grayscale spectrograms.”

should read:

“All 100 AUC were imported into an Excel sheet and the mean was calculated - the model achieved an average AUC of 0.83 for both colored and grayscale spectrograms.”

The scripts used to extract acoustic features, generate spectrogram images, and implement the CNN algorithms, as described herein, are available on https://github.com/uams-tri/ScientificReports2023Paper_PMC10667335_Code under an Apache 2.0 license.

