# Supplementary material for: A machine learning method to process voice samples for identification of Parkinson’s disease
Source: Sci Rep. 2023 Nov 23;13:20615. doi: 10.1038/s41598-023-47568-w (PMC10667335; doi:10.1038/s41598-023-47568-w)
Supplement: Supplementary file 1 — Supplementary Figures. [file 41598_2023_47568_MOESM1_ESM.docx]

Supplementary Figures

#
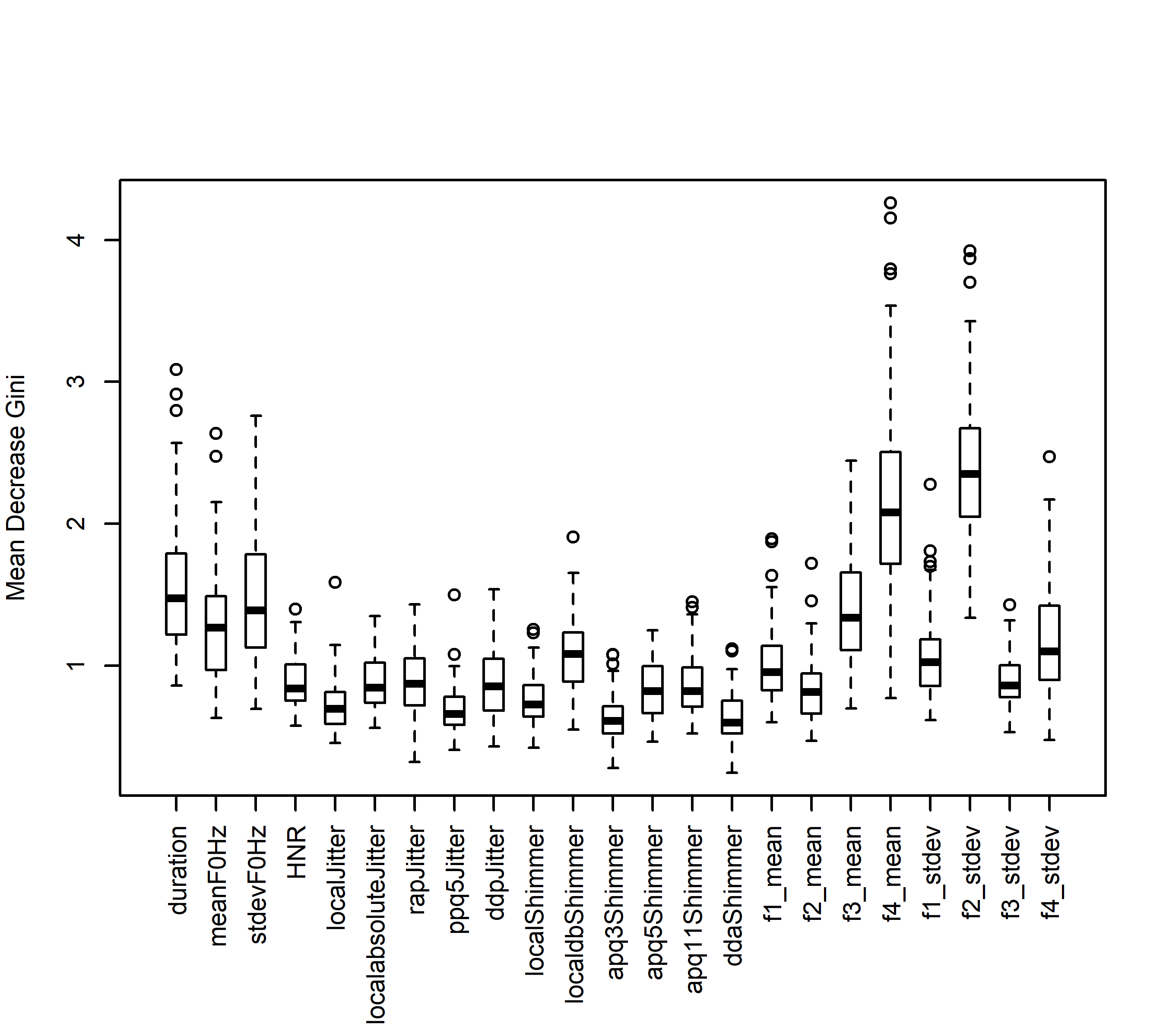


**Figure S1.** Acoustic signal features importance assessed by the mean decrease Gini metric of the random forest classifier.

**
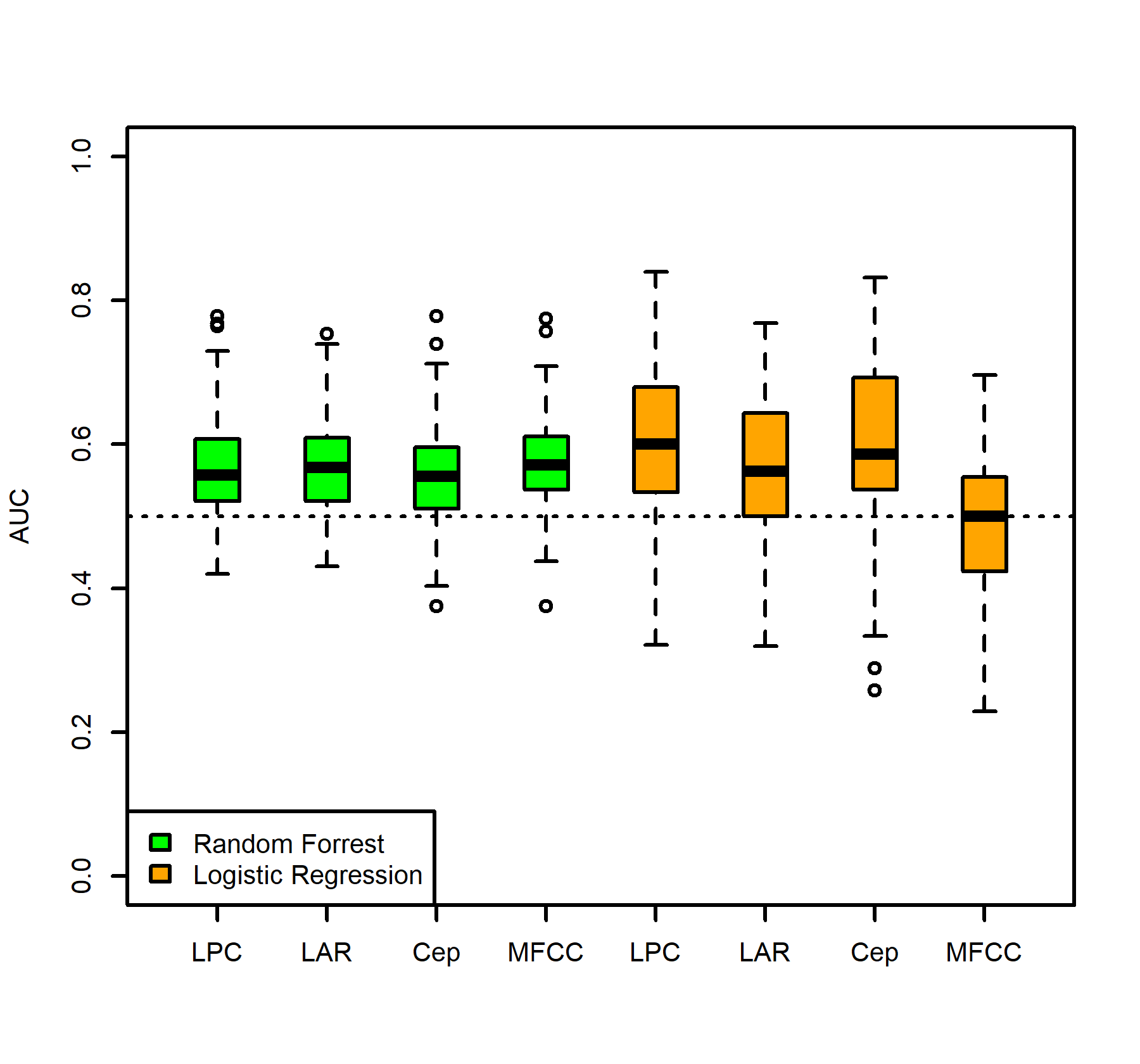
**

**Figure S2**. Estimated classification AUC achieved in 100 iterations using the random forest and logistic regression classifiers with the spectral features mean vectors.


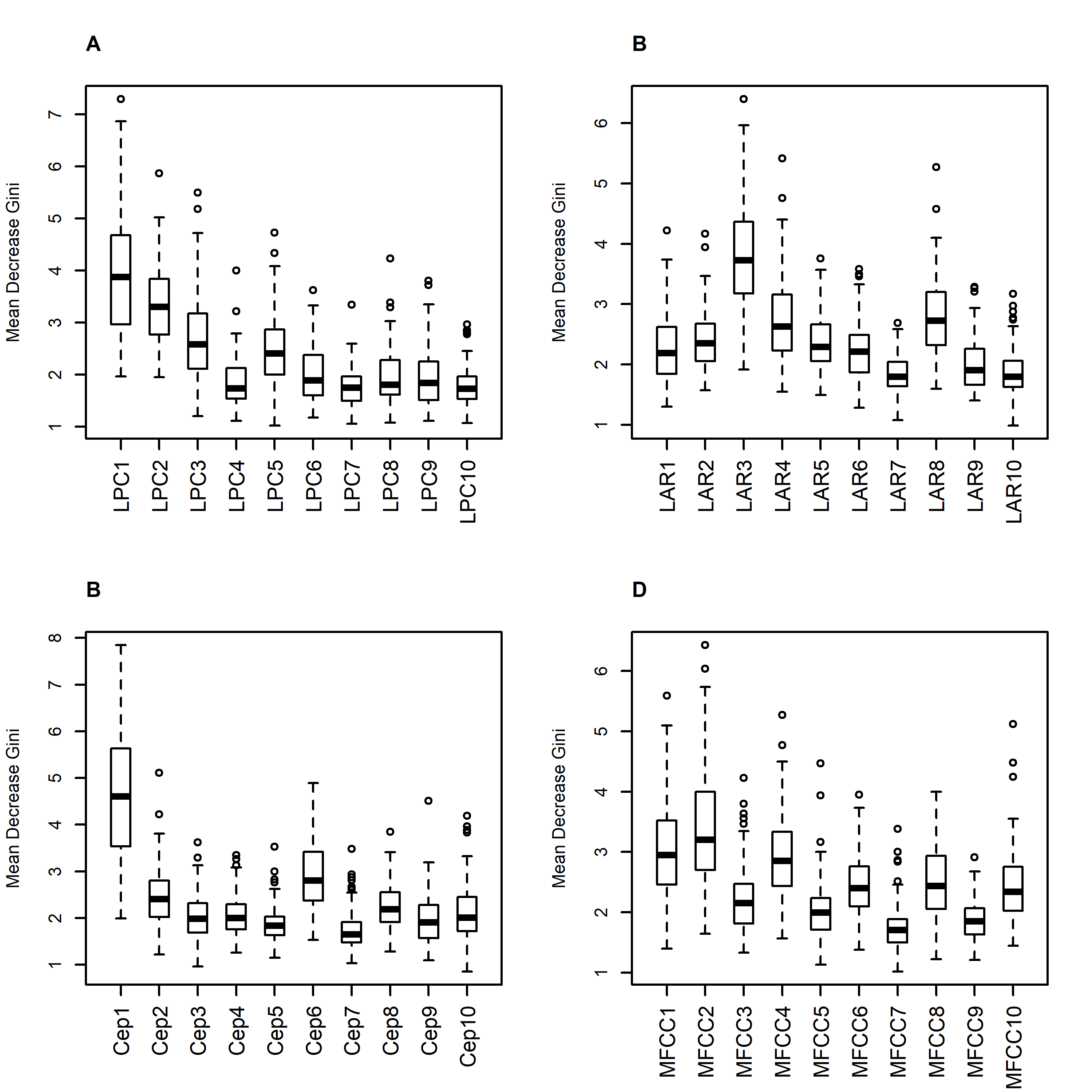


**Figure S3.**Estimated feature importance using the spectral features mean vectors. Importance assessed by the mean decrease Gini metric of the random forest classifier.


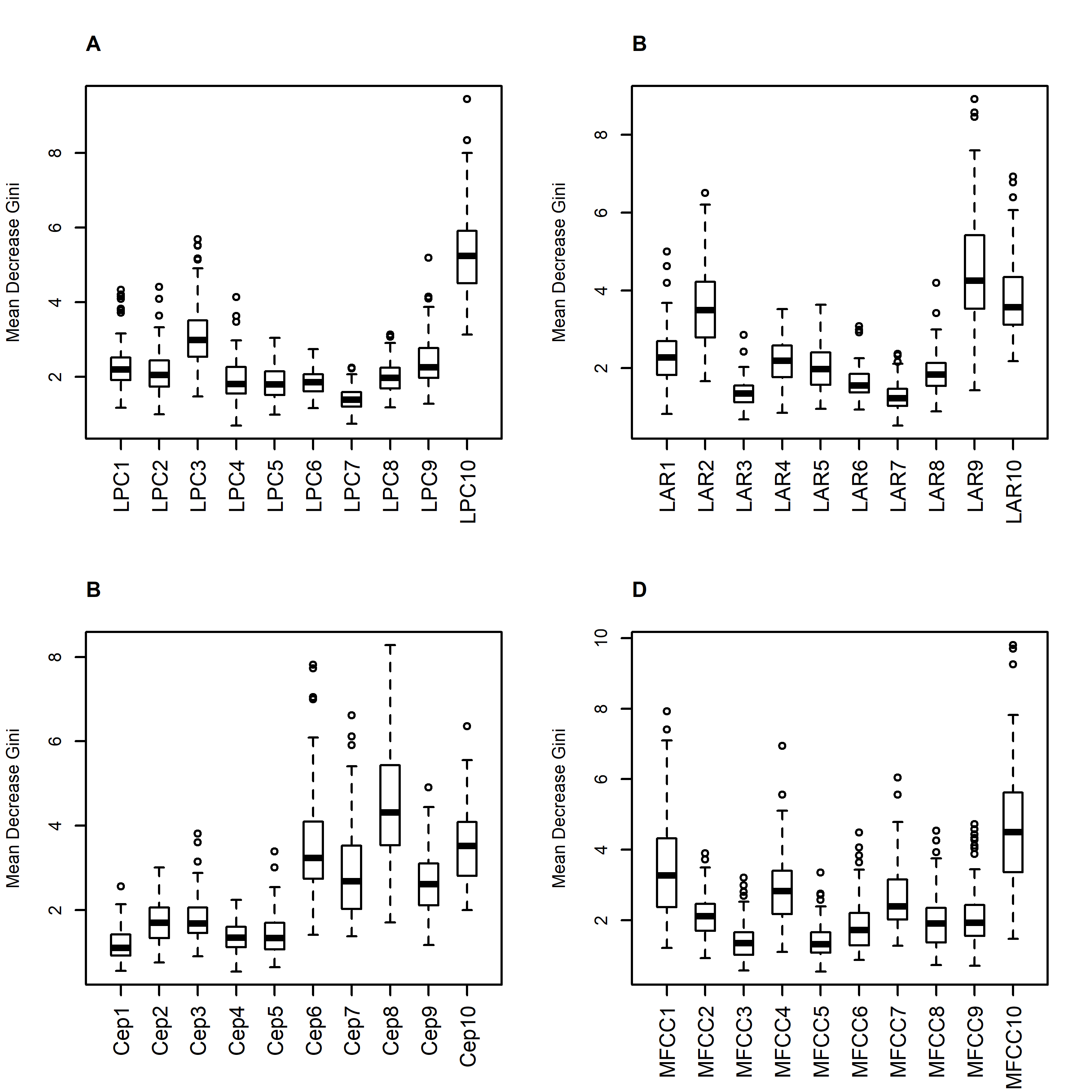


**Figure S4.**  Estimated feature importance using the spectral features variance vectors. Importance assessed by the mean decrease Gini metric of the random forest classifier.
